# Arabic-speaking migrants’ experiences of the use of interpreters in healthcare: a qualitative explorative study

**DOI:** 10.1186/1475-9276-13-49

**Published:** 2014-06-16

**Authors:** Emina Hadziabdic, Katarina Hjelm

**Affiliations:** 1 Department of Health and Caring Sciences, Faculty of Health and Life Sciences Linnaeus University, Växjö, SE-351 95, Sweden; 2 Department of Social and Welfare Studies, Linkoping University, Linköping, Sweden

**Keywords:** Arabic-speaking persons, Focus group interview, Interpreters, Healthcare, Qualitative content analysis

## Abstract

**Introduction:**

Arabic-speaking migrants have constituted a growing population in recent years. This entails major challenges to ensure good communication in the healthcare encounter in order to provide individual and holistic healthcare. One of the solutions to ensure good communication between patient and healthcare staff who do not share the same language is to use a professional interpreter. To our knowledge, no previous qualitative studies have been found concerning Arabic-speaking migrants and the use of interpreters. This study aims to ascertain their individual experiences which can help extend our understanding of the studied area.

**Method:**

A purposive sample of 13 Arabic-speaking persons with experience of using interpreters in healthcare encounters. Data were collected between November 2012 and March 2013 by four focus-group interviews and analysed with qualitative analysis according to a method described for focus groups.

**Results:**

Four categories appeared from the analysis: 1) The professional interpreter as spokesperson; 2) Different types of interpreters and modes of interpretation adapting to the healthcare encounter; 3) The professional interpreter’s task and personal properties affected the use of professional interpreters in a healthcare encounter; 4) Future planning of the use of professional interpreters in a healthcare encounter. The main findings were that the use of interpreters was experienced both as a possibility and as a problem. The preferred type of interpreters depended on the interpreter’s dialect and ability to interpret correctly. Besides the professional interpreter’s qualities of good skill in language and medical terminology, translation ability, neutrality and objectivity, Arabic-speaking participants stated that professional interpreters need to share the same origin, religion, dialect, gender and political views as the patient in order to facilitate the interpreter use and avoid inappropriate treatment.

**Conclusion:**

The study showed that the personal qualities of a good interpreter not only cover language ability but also origin, religion, dialect, gender and political views. Thus, there is need to develop strategies for personalized healthcare in order to avoid inappropriate communication, to satisfy the preferences of the person in need of interpreters and improve the impact of interpretation on the quality of healthcare.

## Introduction

As a result of ongoing global migration and globalization, many societies all over the world have become multicultural, with many migrants who do not speak the official language of the host country. This causes a communication gap in healthcare service that might lead to health disparities [1], inappropriate treatment [2] and longer hospitalization [3]. Previous literature reviews [4-6] and a clinical study [7] have recommended the use of professional interpreters to overcome communication barriers, to improve patient satisfaction, equity and quality of healthcare. Working with an interpreter in a healthcare encounter can provide an enriching opportunity with broader knowledge and perspectives but may also result in decreased privacy in the caring relationship [8].

The mixture of migrants in Sweden has changed in recent years, with an increasing number of people from outside Europe, especially Arabic-speaking persons, and this number is expected to increase in the future [9]. To our knowledge, no previous study has focused on Arabic-speaking migrants’ own experiences of the use of interpreters in healthcare encounters based on qualitative information. A qualitative study gives the opportunity to elicit the person’s individual thoughts, perceptions and experiences in order to obtain a deeper and more complete understanding of the problem area. Thus, it is important to investigate Arabic-speaking migrants’ experiences of the use of interpreters in healthcare service.

When reviewing the literature, previous research focusing on migrants’ experiences of the use of interpreters in healthcare with a quantitative study design concerning Micronesian-speaking migrants in the United States (US) [10] found that they preferred using a family member or friend as interpreter; in contrast, another quantitative study of Arabic-speaking migrants in Sweden found that they preferred using a professional interpreter because of their training, ensuring high-quality language skills, and being employed by an interpreter agency [11]. Previous qualitative studies have focused on migrants’ experiences of the use of interpreters in healthcare, mainly concerning Bengali-speaking migrants in the United Kingdom (UK) [2,12], who preferred a family member or friend as interpreter; Bosnian/Croatian/Serbian-speaking migrants in Sweden [8] and Bosnian/Croatian/Serbian- and Russian-speaking migrants in Ireland [13] preferred using a professional interpreter. However, previous studies have shown conflicting results in the choice of who is best to interpret, with a preference for family members as interpreters, mainly among Bengali migrants in the UK [2,12] and Micronesian-speaking migrants in the US [10]; and professional interpreters regarding Arabic-speaking migrants [11] and mostly Bosnian/Croatian/Serbian-speaking migrants in Ireland [13] and Sweden [8]. Due to these disparities, it is important to investigate Arabic-speaking migrant groups living in Sweden by using a qualitative approach in order to confirm findings from a previous quantitative study and to get a more comprehensive understanding of the problem area.

### Aim

The aim of this study was to explore Arabic-speaking migrants’ experiences of the use of interpreters in healthcare service.

## Method

### Design

A qualitative exploratory study was implemented [14]. Focus groups were used for data collection as the group process enables members to express views that might not have been disclosed in an individual interview [15].

### Participants and procedure

Purposive sampling was used to ensure a range of participants differing in age, gender and educational level and representing different perspectives [15]. The study included adult individuals who had Arabic as their mother tongue, and had experience of using an interpreter in healthcare. The reason for choosing this group was that Arabic-speaking persons from the Middle East constitute the largest non-European migrant group in Sweden [16].

To come into contact with participants the principal investigator contacted representatives of adult education facilities for immigrants by telephone. The representatives were requested to invite Arabic-speaking persons with different backgrounds living in two different cities in a county in Sweden to participate in an information meeting. A time was set for meetings when information (verbal and written in Arabic) was given by the principal investigator (EH) and an assistant moderator who is bilingual with Arabic as mother tongue about the aim of the study, focusing on the use of an interpreter in healthcare encounters from their perspective, about the implementation of the study and the ethical considerations. Written information about the aim of the study, the implementation of the study and the ethical considerations together with a prepaid envelope to return to the principal investigator (EH) were given to voluntary participants so that they could answer when appropriate. The principal investigator’s contact details were included in case the participants had any questions. Those interested in participating sent their address to the first author who contacted them to set a time and place for the interview.

Five men and eight women, aged 21–60 years (median 32 years), with a time of residence in Sweden from two months to 11 years (median 3 years) were included. All were refugees with valid residence permits, mostly born in Iraq, with a high level of education (see Table 1). This is thus, a common picture of the Arabic-speaking population in Sweden [17]. Four focus-group interviews with 13 persons were conducted in two different cities in a county in Sweden, located in an immigrant-dense region.

**Table 1:** Characteristics of the study population

**Variable**	**Foreign-born persons (N = 13)**
Gender *(n)*	
Male	5
Female	8
Age (years)*	21–60 years (median 32 years)
18–28 years	5
29–39 years	4
40–49 years	2
50–59 years	1
60–70 years	1
Length of residence in Sweden (years)*	Two months to 11 years (median 3 years)
Educational level	
Primary school	2
Secondary school	6
University ≤ 2 years	2
University ≥ 2 years	3
Country of birth	
Iraq	5
Syria	4
Lebanon	1
Jordan	1
Yemen	1
Palestine	1

*Values are median (range).

### Data collection

Data were collected between November 2012 and March 2013. A semi-structured interview guide based on a literature review and experiences from previous studies [8,18] was used as a prompt to encourage discussion [15]. The interview guide focused on experiences of using interpreters in healthcare, problems and improvements relating to the use of interpreters. Examples of the key questions were: How do you experience use of interpreters? Please describe a positive and a negative situation where you have used an interpreter. Follow-up questions were: What worked well? What did not work well, and why? What did you think? What did you feel? What did you do? [8,18].

Interviews were held in Arabic and were conducted by the same moderator (first author), with expertise in qualitative methods and migrant issues in health care, and an assistant moderator who is a trained bilingual person with Arabic as mother tongue, broadly familiar with the research topic and the local culture, thereby reducing communication barriers and translating the discussion to reflect the style in which it was conducted. All interviews were held in secluded venues in adult education facilities chosen by the participants. The venues were neutral and quiet, familiar, accessible and easy to find for participants [15,19].

A pilot focus group was held to test the interview guide and the role of the moderator and assistant moderator [15,19]. The pilot focus group turned out well and the data were found to be of good quality and were therefore included in the analysis. The focus group sessions lasted about one hour and included three to four persons. Furthermore, two groups had mixed gender (a group with two women and a man vs. one group with a man and two women) and there was one group each with four female and three male participants.

The interaction in each group was lively, with knowledge transfer, supportive communication, active body language and discussing all topics in the group. The interaction in the focus-group interviews was particularly intensive while discussing interpreters’ dialect. However, this elicited data and had no negative effect on the communication.

Interviews were audio-taped, transcribed verbatim and then translated by the assistant moderator who is bilingual. The translation from Arabic to Swedish and from Swedish to English was certified as accurate by a professional translator [19,20].

### Ethical considerations

Approval for the study was obtained from the Ethics Committee of Linköping University, Sweden. Swedish law concerning the regulation of ethics in research involving persons [21] was followed and the study was implemented according to the ethical principles stated in the Declaration of Helsinki [22] and thus written informed consent was obtained from the participants. To preserve the confidentiality of the data, the tapes and transcripts were anonymized and coded by number. The analysis and presentation of the data were done in a way that concealed the participants’ identity. Participants were assured they could withdraw from the focus groups at any time without explanation. Only the first author had access to all data, which were stored in a locked space at the principal investigator’s (EH) workplace.

### Data analysis

Data analysis was performed as described by Krueger and Casey [15] and was aimed to identify patterns and discover relationships between ideas with the assistance of the qualitative analysis software Atlas Ti (ATLAS.ti Scientific Software Development GmbH). Analysis of data proceeded simultaneously with data collection until no new information emerged.

Firstly, analysis started with summaries directly after the group discussions about what informants had said and the interaction in the group (see Table 2) [15,23]. Secondly, the text from the interviews was read several times to give a broad picture of the data. Thirdly, the text was divided into meaning units, and thereafter coded. Codes with similar meanings were grouped in subcategories according to their differences and similarities. Lastly, comparisons were made during the whole analysis between subcategories and the text as a whole. The process was repeated until no new information appeared. Subcategories with similar meanings were combined into categories [15].

**Table 2:** Example of the data analysis steps

**Category**	**The interpreter as spokesperson**	
Subcategory	The professional interpreter helps to enable expression of concerns, feelings and pain in the mother tongue	The professional interpreter’s presence complicates the healthcare encounter
Codes	Positive experiences of using a professional interpreter	Negative experiences of using a professional interpreter
Meanings units	It is important to use the interpreter in order to help the person to be able to explain feelings, pain and get the information.	It makes it difficult to talk openly about sensitive topics regarding relations and bodily problems.
Quotes	*P 1 (4) “It is really important to use the (professional) interpreter when I cannot speak Swedish… as an interpreter can help you and it is through him I can explain and get the information. It can be very important to have an (professional) interpreter.”*	*P1 (3): “There are some things that I’m ashamed to say in the presence of an* (professional) *interpreter related to my personal stuff… between me and my wife or within the family… It is about the body, … and it is connected with religion and things such as haram.”*
	*P: 2 (4) “To explain what I feel and where I have pain.”*	

Investigator triangulation was used to validate the findings [14]. The first author conducted the analysis and established subcategories. Categories were checked and discussed with the co-author to ensure that she agreed with the interview material. In order to ensure confirmability, verbatim quotations were used to illuminate the results and to verify the categorization [14]. To increase the credibility, analysis proceeded to the point when no new information appeared in order to achieve maximal variation [24]. The result of a study with an exploratory aim depends more on the involvement of the informants in each group and their interaction than on the actual number of informants, as the prime objective is to obtain the maximal variation, as often occurs after three focus-group interviews [15]. Careful descriptions of the method are given to ensure dependability [14].

## Results

**Figure 1:**
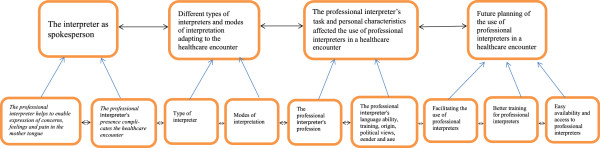
Four categories inclusive nine subcategories emerged from data analysis.

Four categories appeared from the analysis: 1) The professional interpreter as spokesperson; 2) Different types of interpreters and modes of interpretation adapting to the healthcare encounter; 3) The professional interpreter’s task and personal characteristics affected the use of professional interpreters in a healthcare encounter; 4) Future planning of the use of professional interpreters in a healthcare encounter emerged from Arabic-speaking individuals’ experiences of the use of interpreters in healthcare with the respective subcategories (Figure 1). Thus, all four categories were represented in all focus groups and all focus groups exept one had suggestions for improvement of the interpretation situation were given. Informants in the group that had no proposals for improvement were satisfied with the current interpretation situation (see Table 3).

**Table 3:** Example over the number of statements mentioned in each category

**Categories**	**No. of statements’**
The interpreter as spokesperson	70
Different types of interpreters and modes of interpretation adapting to the healthcare encounter	74
The professional interpreter’s task and personal characteristics affected the use of professional interpreters in a healthcare encounter	158
Future planning of the use of professional interpreters in a healthcare encounter	20
Total number of statements	322

### The professional interpreter as spokesperson

#### The professional interpreter helps to enable expression of concerns, feelings and pain in the mother tongue

The use of interpreters was found positive since a professional interpreter gave the possibility for persons who do not speak the same language as healthcare staff to use verbal communication. Informants described confidence being able to express concern, feelings and pain in the mother tongue and the professional interpreter translated their state of health.

*P1 (4)…good to use an* (professional) *interpreter when I do not know the language regardless of what type of care situation it is, if there are two people who cannot communicate with each other … then we need a professional interpreter to be able to translate between each other. … I think it’s really important.*

*P1 (4): … it is best to explain to an* (professional) *interpreter in the language that you know and not explain to the physician in a different language (English) as that can prevent me from saying what I want to say…*

#### *The professional interpreter’s* presence *complicates the healthcare encounter*

The use of a professional interpreter was experienced as a kind of problem or hindrance. The informants described feeling uncertain about whether the interpreters were able to translate their health problems literally and about their own ability to talk openly about sensitive topics regarding relations and bodily concerns in the presence of the interpreter. They explained that it was related to their origin, where people were not comfortable about talking openly about relations and bodily concerns in front of others.

*P1 (3): There are some things that I’m ashamed to say in the presence of an* (professional) *interpreter. It is about the body, I’m ashamed and it is connected with religion and things such as haram* (used to describe what is absolutely forbidden for a Muslim).

Some of the participants were suspicious about the professional interpreter’s ability to be objective if the interpreter did not have the same origin as them. They explained that this was related to serious political conflicts that exist between the Arab countries.

The informants experienced situations with the use of a professional interpreter with the wrong language or dialect, who had limited translation skills and who had not assured confidentiality, which resulted in limited communication. There was uncertainty as to whether a professional interpreter made literal interpretations or not.

The presence of a professional interpreter in a healthcare encounter was described as difficult as it meant not being able to talk directly to healthcare staff. Communication through a professional interpreter took longer because the information needed to be provided twice.

P1 (4): … to explain to the professional interpreter what you feel and where you have pain and the interpreter may not be able to explain, express him/herself and translate all the words that I want him/her to do. It is easier when I talk directly to a doctor who knows the language and the doctor himself understands the subject without the use of an interpreter … (in the presence of a professional interpreter) I have to explain and tell the interpreter everything before I start talking about the main issue.

Problems were also reported concerning healthcare professionals’ unwillingness to book a professional interpreter even when they know about the patient’s communication difficulties, and this led to the use of family/friend/bilingual healthcare staff as interpreters. Another factor that was experienced as problematic was lack of documentation in the referral record that the person needed a professional interpreter. This resulted in the consultation being cancelled.

P1 (1): no, when healthcare staff notice that I know some Swedish, in those cases they do not use the interpreter any more …

*P2 (3): I am new in the country and believed that it was their* (healthcare staff) *responsibility to book an interpreter … They* (healthcare staff) *usually do not order an interpreter for me even they know that I can’t speak Swedish, so I use a friend as the interpreter.*

*P1 (3): … I had a referral from the health centre, and nursing staff at the health centre knew that I need an interpreter but they did not write it on the referral, so when I came in to the doctor in the hospital the doctor asked if I needed an interpreter and I said yes and then they* (healthcare staff) *just cancelled the visit.*

### Different types of interpreters and modes of interpretation adapting to the healthcare encounter

#### Type of interpreter

The preferred type of interpreters depended on the interpreter’s use of dialect and ability to interpret correctly. Informants have indicated that the use of a professional interpreter enhanced the possibility of receiving adequate treatment. Their education, together with high-quality language competence and skill in medical terminology and ability to keep the code of confidentiality, helped participants to trust in good interpretation.

*P2 (1): … prefer an* (professional) *interpreter to interpret at all important healthcare encounters…*

*P3: (1) the* (professional) *interpreter must have experience so that they can interpret accurately and they must be trained and always controlled …*

P2 (3): when it comes to important issues such as when I can risk life, I prefer a professional interpreter.

P1 (3): … prefer a professional interpreter for all healthcare situations because in those cases it is important to interpret correctly about the medical concepts, it is important to get the right translation.

Using family members as interpreters was preferable in uncomplicated care situations such as when patients had a cold. Other advantages of using family members as interpreters were feeling security and trust in the family member, family members were perceived as being familiar with patients’ concerns and they were easily available. During unexpected visits they seldom had access to professional interpreters and in those cases the family member was used as interpreter. This can also be perceived as a disadvantage because of incomplete translation, family members not having the duty of confidentiality (or even knowledge about it) and having limited language skills. Further, patients might feel ashamed of some matters in the presence of family members and thus not be willing to convey a message, for example, in the presence of family members of the opposite sex. The use of children as interpreters was found inappropriate because of their limited language skills in medical terminology.

*P2 (3): … situations which are easy to handle such as when I have a cold … it is okay to use a family member as interpreter … and in cases if there is no* (professional) *interpreter then it’s okay to use the family as an interpreter.*

*P3 (4): it is much easier to use my husband as an interpreter because we live together … and he is familiar with my condition and then I do not need to repeat the information to an* (professional) *interpreter again. … He* (my husband) *… has language competence.*

*P3 (1) … it can be negatively affected with the use of … a friend who interprets about my personal issues …Then maybe she* (the friend) *will spread to others what I have said, …*

*P2 (3): It is not good … because they* (family) *can’t interpret correctly regarding the medical concepts that need to be translated correctly.*

P1 (3) It is not good to use a child as interpreter… it is not good for a child does not know enough language.

Concerning the use of bilingual healthcare staff as interpreters, contrary perceptions were expressed. On one hand positive conceptions as regards many practical and financial benefits and on the other hand negative conceptions such as that bilingual staff could show a superior attitude, do not have duty of confidentiality, and interpreting is not part of their task, which could affect interpretation negatively.

P1 (3) It is better to use healthcare staff as interpreters because there’s no need to pay for an interpreter …

*P2 (3).* (The negative side may be) *if the nurse or the doctor does not have time to interpret, and if they* (healthcare staff) *are not permitted to interpret.*

#### Modes of interpreting

Face-to-face interpretation was for most people the preferred method of interpretation in healthcare. Participants said that face-to-face interpretation allowed observation of the interpreter’s verbal as well non-verbal language. Interpreters on the spot were perceived as having good language skills and translation ability.

P2 (3). A professional interpreter on the spot, I would like to have when visiting the doctor because there are certain things you explain through your body language so that the interpreter can better understand.

Telephone interpretation was the desirable method in uncomplicated care situations. The benefits of telephone interpreters were that they are easily accessible and allow participants to be more anonymous when talking about sensitive matters and/or having physical examinations, especially with a professional interpreter of the opposite sex. There were also negative perceptions about the use of telephone interpretation, as participants usually found that the telephone interpreter did not share the same dialect as the patient, which resulted in inadequate understanding of the information and ultimately in inadequate treatment. Furthermore, informants described the use of telephone interpretation as problematic in terms of a lot of noise when all parties speak at the same time and technical problems such as impaired hearing. Interpretation by telephone was the most commonly used form of interpretation for unexpected visits.

*P3 (1): … advantages of using a telephone interpreter … a woman who is ashamed to talk about certain diseases … by an* (professional) *interpreter who is a man … in emergency visits, only telephone interpreters are used …*

P1 (3): Emergency cases, because you need to get some interpreter quickly and directly it is helpful to use a telephone interpreter … I had an interpreter by telephone … I explained to the telephone interpreter that I am so old that I can’t lose weight so easily, for the fat is attached mostly to the stomach, the telephone interpreter had interpreted that I’m hard in the stomach (constipated) and the doctor gave me medicine that makes your stomach loose. So it is good to have the interpreter on the spot.

P2 (2) it is not good to have telephone interpreters because I can’t hear well over a phone.

*P (3): The* (interpreter’s) *dialect may be difficult to understand through the telephone.*

### The professional interpreter’s task and personal characteristics affected the use of professional interpreters in a healthcare encounter

#### The professional interpreter’s profession

The professional interpreter’s task was described as interpreting everything in an encounter, even regarding a cancer diagnosis.

*P2 (3): he* (professional interpreter) *should only interpret and nothing more. … should not be delayed, and should interpret correctly. … The interpreter’s task is to interpret and not to lie to the doctor even if the patient wants the interpreter to lie to the doctor … An interpreter should not lie because he is sworn to keep the code of confidentiality. The interpreter’s profession is only to interpret …*

Some informants described how a professional interpreter could also have an important task as a practical and informative guide in the healthcare system, thus helping them. The task could also include informing the patient about rules and procedures applicable in the host country in order to adapt more easily to a new culture. Others approved the current policy of providing a translation between two languages in a healthcare encounter, so that the interpreter’s task as a practical and informative guide could be performed on a voluntary basis.

(4): … *I think that he* (the professional interpreter) *should not only interpret at the healthcare encounter. The* (professional*) interpreter should also help me with something more when I need it, such as explaining things…*

#### The professional interpreter’s language ability, training, origin, political views, gender and age

It was emphasized that a professional interpreter who shared the same language and dialect as the individual in need of an interpreter was most important for the individual to be able to understand and to be understood. Participants described how using the interpreter with a different dialect than themselves resulted in inadequate communication. Other important personal factors in the professional interpreter were skills in languages and medical terminology, translation ability, education, having the same origin and political affiliation as the person in need of an interpreter.

*P2 (1)…* (professional) *interpreters … who do not have the same dialect … I do not understand what the interpreter is saying and … the* (professional) *interpreter does not understand me when I talk, … prefer a* (professional) *interpreter from the same country as me because I can understand better …*

P3 (1): Since there are disputes between Arab countries which mean that I do not rely on an interpreter from another country because of political differences between the countries and religion with the result that the (professional) interpreter does not to interpret objectively …

*P2 (4): a* (professional) *interpreter should be competent in mother tongue, … and Swedish … should be able to explain in both languages … should be trained and use academic language.*

The professional interpreter’s gender was relevant especially in relation to sensitive matters such as physical and/or gender examination. They explained that it had to do with cultural and religious issues related to their origin where the people were not comfortable with such subjects. Some of the male participants stated that female professional interpreters were a better choice than male professional interpreters for female patients and children because they were more familiar with subjects related to being a woman or a child.

*P2 (4): in cases concerning a woman who is on a visit to a doctor then it’s better to have a female* (professional) *interpreter. We come from the east, we have a different culture … and it feels more comfortable with a female interpreter. And … she* (interpreter) *has competence in medical terms because she is a woman herself … a woman knows about child issues more than a man… then it may be that a male* (professional) *interpreter has difficulty interpreting because he can’t understand the mother and interpret correctly for her.*

The age of the professional interpreter was considered an important personal property because it is relevant to the professional interpreter’s language skills and translating ability. However, some participants described how a professional interpreter who was young had better language skills and translating ability, and others said that professional interpreters who were older or of the same age as the participant had better language skills and translating ability.

*P3 (1): … that he* (professional interpreter) *should be young and be competent in the medical language … for the older* (professional interpreters) *can’t use the modern language that young people use …*

*P2 (4): … for just a younger* (professional interpreter) *can understand me better because we’re the same age … Then they* (older professional interpreters) *can be a disadvantage for me because the elderly can’t translate quite like a younger* (professional interpreter) *can.*

*P2 (3): if he* (professional interpreter) *is older then he is already trained and has special training for professional interpreters.*

### Future planning of the use of professional interpreters in healthcare encounters

#### Facilitating the use of professional interpreters

To facilitate the use of professional interpreters, it was important to have trust in the professional interpreter’s ability to transfer information correctly. This could be done by previously knowing the interpreter, being able to choose a professional interpreter by themselves and the type and mode of interpretation. Further, healthcare professionals contact the professional interpreter themselves or suggest to the patient an interpreter whom they find good, and healthcare professionals should be aware of their own use of language for the interpreter’s understanding and translating to the patient.

P3 (1): I would choose for myself the type of interpreter I prefer … there must be more options of different interpreters to access and then I can choose which one I want.

*P2 (3): if the doctor has a different Swedish dialect and the doctor also is not Swedish, then it will be also difficult for an* (professional) *interpreter* (to understand)*. So it is not only the interpreter’s dialect, but the doctor’s dialect is also important because if the* (professional) *interpreter can’t understand the doctor’s dialect then the* (professional) *interpreter can’t explain to the patient … There are some doctors who use the same* (professional) *interpreter because doctors are familiar with using particular (professional) interpreters and then it is important for the doctor to have the opportunity to call and book a particular* (professional) *interpreter.*

Informants wished that healthcare professionals would strive for consistent use of a professional interpreter who had the same language, dialect and origin of country as the patient. In cases where the professional interpreter and the patient do not share the same dialects, it was proposed that professional interpreters should use the patient’s dialect or Modern Standard Arabic.

*P2 (4):* (healthcare professionals) *should take into account that they book an interpreter with the right language and dialect for me. It is easier to understand an interpreter who speaks the same language, dialect and who is from the same country.*

P2 (1): the professional interpreter should use the standard Arabic language so that the (professional) interpreter and the Arabic-speaking person understand each other.

#### Better training for professional interpreters

One way to improve the use of professional interpreters is to improve the training of interpreters, both in general and in language skills. Another way was to improve the professional attitude of interpreters, such as the ability to keep the code of confidentiality.

*P1 (1): It is important to have a code of confidentiality. There are many* (professional interpreters) *who say it but then spread the information anyway.*

*P3 (1): they* (professional interpreters) *must be trained. Better education so that not just anyone can be an interpreter.*

#### Easy availability and access to professional interpreters

Participants wished that professional interpreters should be employed in hospitals, in order to ease availability and access to professional interpreters.

P3 (3): I suggest that every hospital employ two or three professional interpreters directly at the hospital and then use them as permanent interpreters.

## Discussion

This study provides important new data on issues faced by Arabic-speaking migrants who use interpreters in healthcare, often excluded from research because of their inability to speak the language of the host country. The use of interpreters was experienced as a benefit as it gave the possibility to express concerns, feelings and pain in the patient’s mother tongue. However, it was also experienced as problematic because of feelings of insecurity as to whether the interpreters were able to translate correctly, and the feeling of shame about openly talking about relations and bodily concerns in the presence of the interpreter. The preferred type of interpreter depended on the interpreter’s use of dialect and ability to interpret correctly. The professional interpreters’ role was to translate literally and objectively, with the qualities of a good knowledge of languages and medical terminology, good translation ability, sharing the same origin, religion, dialect, gender and political views as the patient. Healthcare staff should strive for consistent use of a professional interpreter with the same language, dialect and origin as the patient in order to facilitate interpreter use.

A new finding, not described previously, was feelings of insecurity about whether the information was correctly interpreted and of shame about talking openly about relations and bodily concerns in the presence of a professional interpreter with a different dialect. Even among those speaking the same language, e.g. Arabic, but coming from different countries, there might be differences related to dialects that are not mutually intelligible [25] as Arabic-speaking persons show their geographic origin, social class, country of origin, rural versus urban upbringing and education through the pronunciation of words [26]. Regional differences in Arabic dialects are great enough to create major misunderstanding in a healthcare encounter, which makes it difficult to utilize a professional interpreter service broadly. Thus, the healthcare staff need to consider the interpreter’s language and also dialect when ordering a professional interpreter and the interpreting agency needs to meet their demands in order to provide accurate communication and understanding. Another factor to be aware of in the interpretation situation is the professional interpreter’s gender. Previously [27] it has been shown that the person’s gender is significant for the integrity of a patient; the subject of sexuality and bodily examinations is considered especially private and embarrassing for the majority of Arabic-speaking female patients, and consequently they prefer to be seen by a female physician [28]. This study found that some of the Arabic-speaking persons may feel more comfortable talking about their personal medical issue with an interpreter of the same sex. Therefore, it is important to arrange the interpretation situation so that the professional interpreter is of the same gender as the Arabic-speaking patient in order to improve the impact of interpretation on the quality of healthcare.

A new finding compared to our previous quantitative study among Arabic-speaking migrants in Sweden [11] was that participants described healthcare professionals’ unwillingness to book a professional interpreter even when they know the patient’s communication difficulties. However, this finding confirms previous qualitative studies among Bosnian/Croatian/Serbian-speaking migrants in Sweden [8] and families [29]. This could be explained by the differences in the methods used; the use of qualitative methods allowed the researcher to ask open-ended questions and to probe for any understanding of the person’s individual thoughts, perceptions and experiences which could not be grasped in a structured survey with close-ended response alternatives determined by the researcher [14].

When talking about the preferred type of interpreter, the findings of this study showed that there was no homogeneous picture; participants stated that it depends on the interpreter’s use of dialect and ability to interpret correctly. This finding was in contrast to data from previous studies [2,8,10-13] showing dissimilarities between different migrant groups, preferring different kinds of interpreter. Using professional interpreters gave benefits such as their ability to interpret literally, objectively and without having any relation to the patient [8,18]; the use of family members as interpreters led to positive feelings of security and trust [2,30,31]; and the use of bilingual staff healthcare staff had several positive practical [8,32] and financial advantages [33,34]. However, the use of family and bilingual healthcare staff as interpreters has been associated with poor and problematic interpretation situations because of non-neutrality and confidentiality problems [13,29] and interpretation errors caused by their limited knowledge of medical terminology and inadequate language skills [18,34]. As previously documented, each culture defines or provides religious values and beliefs which often greatly influence human behaviour, shaping one’s view of the world, death and the health-disease process [28]. In some cases healthcare staff and patients can differ in their understanding of illness, preferences concerning treatment and communication styles [28,35]. This study confirmed previous findings that the choice of interpreter was an experience in which cultural beliefs, religious and traditional values are highly relevant for migrant patients. The dissimilarities between different migrant groups challenge recommendations based on previous literature reviews [4-6], and a clinical study [7] which has advised the use of professional interpreters. This study highlights how healthcare should be personalized to the preferences of the individual for the type of interpreter.

This study found that Arabic-speaking individuals preferred to use face-to-face interpretation in healthcare encounters as this allows observation of the professional interpreter’s non-verbal language together with the verbal language, in accordance with previous quantitative studies of Arabic-speaking migrants [11]; previous qualitative studies of Bosnian/Croatian/Serbian-speaking migrants in Sweden [8] and Bosnian/Croatian/Serbian- and Russian-speaking migrants in Ireland [13]. Despite this preference, interpretation by telephone or using the family members as interpreters is still reported as the most frequently utilized method for unexpected visits. Healthcare staff need to be aware when planning communication through an interpreter that the use includes not only verbal language but also that the non-verbal communication plays an important role in the transmission of messages [28,36].

That the professional interpreter has the same political views as the patient was associated with trust in the professional interpreter, and has not been described in other studies. The relational difficulties involved in working with interpreters in healthcare settings have been previously found [37] to be related to issues of trust, control and power. Patient-centred communication and provider/interpreter collaboration has often been missing, with communication tending to be partial and healthcare-staff-centred, with healthcare staff directed their communication towards interpreters, spoke in long turns and asked several questions at once [38]. Some guidelines recommended that healthcare staff speak directly to patients, talk slowly in moderated sequences in order to create good communication for the interpreter so that they may translate clearly all that is said [36,39]. Previously, the role of interpreter has been described as that of a translation machine providing accurate and neutral communication [40] and the role has been questioned because the interaction between the interpreter, patient and healthcare staff is symbiotic and not dyadic in the interpretation situation [41]. However, this study described the qualities of a professional interpreter, in accordance with a previous quantitative study of Arabic-speaking persons in Sweden [11], previous qualitative studies from mainly Bengali-speaking persons in the UK [12] and Bosnian/Croatian/Serbian-speaking persons in Sweden [8] which have been shown to cover the same aspects of language, language ability, interpreter’s character and attitude. This highlights the need to offer professional interpreters who remain neutral, impartial and confidential, convey information, ensure understanding and accuracy [42] irrespective of age, ethnic and cultural origin and geographic or regional differences, in order to ensure effective, accurate and transparent communication which also improves healthcare outcomes.

The strength of this research is that interviews in focus groups with an Arabic-speaking assistant moderator allowed the participants to respond in their mother language to participate and express their views, which increased their comfort level and also helped to maximize the quality of data [15,20]. This gave the opportunity to deepen our understanding of findings from a previous quantitative study [11]. The similarity between the informants’ backgrounds may also have had the result that participants felt confident in expressing their views, which gave the second strength of the chosen method that also ensured a typical picture of the Arabic-speaking population in Sweden [17], whose duration of residence reflected their experiences of the use of interpreters from novice to an expert (11 years). The ability to communicate in the group was supported by the openness of informants while discussing sensitive topics such as the interpreter’s dialect. The third strength of the study was that focus groups functioned well with the study population through the good interaction and group climate during the interviews. Finally, the fourth strength of using focus groups to collect data is that the interaction in the group contributes to the participants’ understanding of the issue discussed and allowed them to provide information that might not have been expressed in an individual interview [15]. Thus, participants’ discussion about of their positive and negative experiences led to increased awareness to avoid in subsequent interpretation situations in order to improve the use of professional interpreters and thus improve communication. Through the focus group conversations a person’s thoughts, opinions and perceptions, even those not completely conscious, can be disclosed [15].

One limitation of this study may be that the procedure of contacting adult education facilities for immigrants to recruit participants could be that individuals who did not attend these services were not included in the research. However, at the information meetings, the participants were also initially asked if they knew others who met the study criteria who could be recruited to the study in order to attain richness in data. Written information in Swedish and Arabic together with a prepaid envelope was given to participants who identified any person who met the study criteria. Thus, non-informants were recruited in this way.

Another limitation may be related to the difficulties of running a mixed-gender focus group e.g. the peacock effect [15]. Having men and women in the same focus group might lead to limited possibilities for women to express their views, as men have a predisposition to speak more frequently and with more power [15]. However, the interaction in the group was lively; all expressed themselves in an equal way and after analysis of four groups there were no signs of any peacock effect.

The assistant bilingual moderator translated the interviews, which could have influenced the findings in both a positive and negative way as the language differed from that of the researchers. The positive way was that the bilingual person was introduced to the study process and had a similar background to the participants, which resulted in the use of more appropriate language to explore the concepts, expressions, ideas and issues discussed [19]. The negative way could be the use of translated data [20], but the translation was checked by a professional translator [19,20]. Regardless of these limitations, this investigation confirms previous quantitative findings from a migrant group with the same cultural and language background [11] and qualitative findings from another migrant group with a different cultural and language background [8]. Furthermore, our study findings are contextual and provide a deeper understanding of the studied topic, and the results can be transferred to other contexts with similar characteristics, as several individuals described a similar picture of their experiences [15].

## Conclusion

This study is the first to explore Arabic-speaking migrants’ experiences of the use of interpreters in healthcare service, which is a growing migrant population internationally and increasingly accessing the healthcare system. The findings highlighted demands to have a professional interpreter on the spot, with the same country of origin, religion, dialect, gender and political views as the patient, in order to avoid communication barriers and to improve the impact on quality of healthcare.

The implication of the study is that, when arranging for the use of professional interpreters in the healthcare sector, it is important to consider that the qualities of a good interpreter not only cover language ability but also origin, religion, dialect, gender and political views. Thus, the healthcare must be tailored to personalized healthcare in order to avoid inappropriate communication, leading to personalized use of interpreters and improving the impact of interpretation on the quality of healthcare.

## Competing interests

The authors declare that they have no competing interests.

## Authors’ contributions

EH was responsible for the study conception and design, development of the interview guide, carried out the data collection, performed qualitative analyses and drafted and revised the manuscript. KH helped in the design of the study and coordination. EH and KH performed the drafting of the manuscript. Both authors read and approved the final manuscript.
